# The effect of exercise rehabilitation with exergames combined with ice therapy in the treatment of obese patients with gout: protocol for a clinical trial

**DOI:** 10.1186/s13063-024-08237-z

**Published:** 2024-06-21

**Authors:** Manting Cao, Hazwani Ahmad Yusof, Jianer Chen, Mohd Faizal Jalil, Siti Khairizan Rahim, Mohamad Zulfadhli Abdullah

**Affiliations:** 1 Department of Community Health, Advanced Medical and Dental Institute, Universiti Sains Malaysia, Kepala Batas, Pulau Pinang 13200 Malaysia; 2 Department of Rehabilitation, Third Affiliated Hospital of Zhejiang Chinese Medical University, Mogan Road, Hangzhou, 310005 China; 3 Department of Clinical Medicine, Advanced Medical and Dental Institute, Universiti Sains Malaysia, Kepala Batas, Pulau Pinang 13200 Malaysia; 4 Internal Medicine Unit, USM Bertam Medical Center, Universiti Sains Malaysia, Kepala Batas, Pulau Pinang 13200 Malaysia

**Keywords:** Gout, Exergames, Ice therapy, BMI, Randomized controlled trial

## Abstract

**Background:**

Gout remains a leading cause of inflammatory arthritis worldwide, and the main risk factor for gout is persistent hyperuricemia. The clinical management of gout is mostly drug-based, and other treatment options are often ignored. This research proposal will explore whether exergames combined with ice therapy can help patients with gout to lose weight, relieve pain, improve the range of movement, improve quality of life, decrease uric acid level, decrease kinesiophobia and improve mental health of patients with gout.

**Methods:**

This experiment will use a two-arm randomized controlled design. The study setting is at the Advanced Medical and Dental Institute (AMDI), Universiti Sains Malaysia (USM). Obese patients with gout (*N* = 30) will be randomly assigned to the control group (receive an exergames intervention) and intervention group (receive an exergames intervention combined with ice therapy). The outcomes measurement will be conducted before (baseline) and after intervention (4 weeks). Then, it will be followed up at 12 weeks.

**Discussion:**

To our knowledge, no study has investigated the effect of exergames and ice therapy among gout patients. This study is expected to demonstrate that exercise rehabilitation facilitated by exergames with ice therapy is more effective in gout management compared to a conventional rehabilitation intervention.

**Trial registration:**

Chinese Clinical Trial Registry (ChiCTR2300070029). Registered on 31 March 2023.

## Introduction

### Background and rationale {6a}

Gout remains a leading cause of inflammatory arthritis worldwide, with persistent hyperuricemia being the main risk factor [1]. Hyperuricemia predisposes individuals to monosodium urate (MSU) crystal deposition in and around the joints [2]. Reducing inflammation and controlling symptoms are the most important intervention goals in gout patients [3]. The prevalence of gout is rising globally [4]. Obesity and aging are two risk factors significantly associated with the development of gout [5]. Therefore, the global rise in gout is considered more prevalent among aging populations and those with higher obesity rates [6]. Dasgupta et al. [7] conducted a study to investigate risk factors for recurrent gout attacks in Malaysian patients, and the findings showed that gout recurrence rates were 44% and 70% in non-obese and obese patients, respectively.

Exercise can effectively manage obesity associated with gout [8]. Additionally, regular and moderate exercise can produce measurable anti-inflammatory effects, reducing the annual frequency of gout attacks, alleviating joint swelling, and lowering pain scores in gout patients [9]. Although exercise is significant for patients with gout, joint pain and swelling caused by the deposition of uric acid crystals in the joints may reduce patients’ adherence to exercise. One strategy to promote exercise adherence in gout patients is the use of exergames. Exergames are technology-driven physical activities that integrate interactive gameplay into the exercise process [10]. Several studies have examined and demonstrated the benefits of exergaming, indicating the potential of using exergames as a viable intervention for increasing physical activity among the sedentary population [11].

Recovery from exercise is essential for a successful exercise intervention [12]. To avoid exacerbating joint inflammation caused by exercise, practitioners may want to consider treating gout patients with ice therapy after exercise. The application of ice therapy after exercise is reported to be beneficial in reducing muscle temperature and blood flow, thereby inhibiting the local inflammatory response [13]. Moreover, numerous studies indicate that ice therapy can alleviate delayed onset muscle soreness (DOMS) within 24 h following exercise [14]. Therefore, for gout patients who are prone to giving up exercise due to fear of worsening pain, ice therapy is a potential method to reduce patients’ kinesiophobia. In addition, the British Society for Rheumatology Guideline for the Management of Gout also suggests that ice could be an adjunct to acute gout treatment, as it can effectively reduce the symptoms in gout patients [1].

The prevalence of gout is showing an upward trend, and many patients’ symptoms are not well controlled [15]. Research has shown that despite the possibility of early diagnosis and understanding of risk factors, most patients continue to relapse after treatment and are at risk for further joint damage and other complications [16]. When asked about the adequacy of gout management and treatment, medical practitioners generally believe that treatment is adequate, while some patients believe it is not sufficiently managed [16].

### Objectives {7}

The clinical management of gout is primarily drug-based, and other treatment options are often ignored. This research aims to explore whether exercise using exergames combined with ice therapy can help patients with gout to lose weight, relieve pain, improve the range of movement, improve quality of life, decrease uric acid levels, decrease kinesiophobia, and improve the mental health of patients with gout. This potential gout management treatment is novel, economical, and easy to access.

### Trial design {8}

**Fig. 1:**
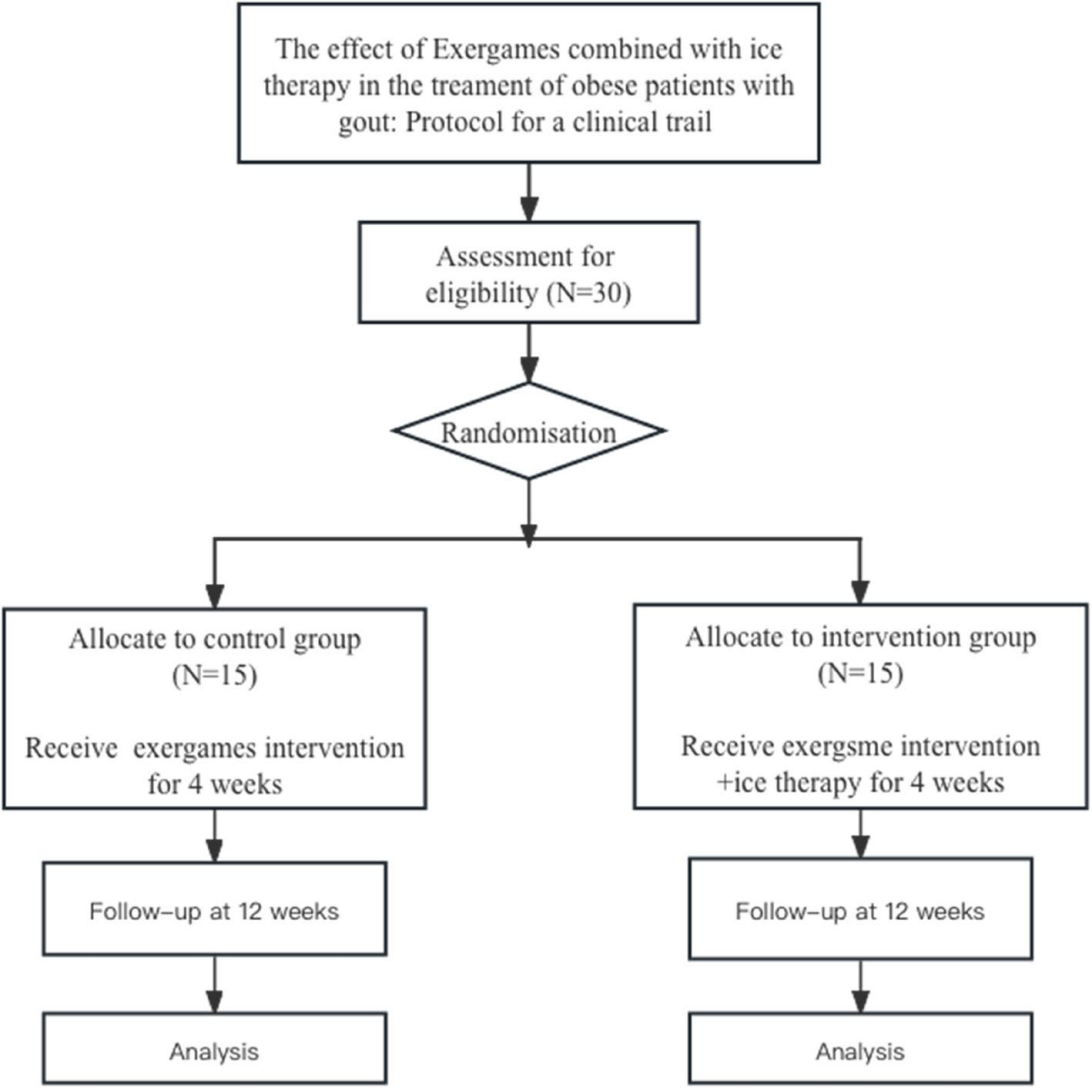
Flow chart of study

This study is a single-blind, single-center, parallel-group, two-arm randomized controlled clinical trial. Thirty patients with gout who meet the inclusion criteria will be randomly assigned in a 1:1 ratio to the intervention group (exergames intervention, *n* = 15) and the control group (exergames + ice therapy, *n* = 15). Outcome measurements will be conducted before and after the intervention and followed up at 12 weeks. The result measurer will be blinded to group allocation. The framework of the study is exploratory. The flow chart of the study is shown in Fig. 1.

## Methods: participants, interventions, and outcomes

### Study setting {9}

The team members of the experiment include the lead investigator, who will record and organize measured data; a physical therapist, who will perform exercise rehabilitation and ice therapy on the participants; a therapist, who will supervise the intervention to ensure uniformity and standardization of treatment; an outcome measurer, responsible for outcomes measurement; and a gout expert, who will provide professional clinical judgment and respond to emergencies during treatment. The study will be conducted at the Advanced Medical and Dental Institute (AMDI), Universiti Sains Malaysia (USM). The participants will register under the exercise clinic, AMDI USM, Malaysia.

### Eligibility criteria {10}

Participant selection will be conducted by attending professionals. The diagnosis of gout refers to the 2015 Gout Classification Criteria developed by the American College of Rheumatology (ACR) and the European League of Associations for Rheumatology (EULAR) [17].

#### Inclusion criteria

The participants must:

1. Have ages ranging from 18 to 70
2. Have BMI ≥ 25
3. Can participate in treatment independently
4. Live in the local area, will not travel far during treatment
5. Meet the preliminary criteria for classifying the acute arthritis of primary gout

#### Exclusion criteria

The participants will be excluded if they are:

1. Patient with the current of urate-lowering therapy (ULT)
2. Patients without gout-related pain or with mild pain
3. Pregnant woman
4. Patients with serious heart or vascular disease
5. Patients with mental problems or a history of mental illness
6. Not meeting the preliminary criteria for classifying the acute arthritis of primary gout
7. Patients who do not meet obesity criteria
8. Patients have a serious medical condition that precludes exercise intervention

### Who will take informed consent? {26a}

Subjects participating in the study will be recruited from ADMI clinics, and advertisements containing detailed research information will be disseminated through ADMI clinic physicians and social media. Before study participation, all subjects will be required to sign an informed consent form. The procedure for obtaining informed consent will be conducted by two experienced physicians (S. K. R. and M. Z. A.) at the ADMI clinic. This procedure will include providing potential participants with the informed consent form and allowing them sufficient time to read the entire document. Additionally, the physicians will thoroughly explain the trial’s interventions, objectives, potential risks, and benefits.

### Additional consent provisions for collection and use of participant data and biological specimens {26b}

This study will request consent to collect participants’ blood samples to assess fasting blood glucose, total cholesterol, triglyceride, high-density lipoprotein (HDL) cholesterol, low-density lipoprotein (LDL) cholesterol, urea, creatinine, estimated glomerular filtration rate (eGFR), and uric acid levels.

### Interventions

#### Explanation for the choice of comparators {6b}

The aim of this study is to evaluate the efficacy of exergames combined with ice therapy, using exergames alone as the comparator. Innovative exergames hold promise for engaging participants in increased physical activity and may offer benefits for highly sedentary individuals or those averse to conventional exercise modalities [18]. Previous studies have shown that exergames have significant effects in reducing BMI, increasing physical activity levels, improving cardiovascular risk factors, and enhancing psychological health [19, 20].

#### Intervention description {11a}

##### Control group

Participants in the intervention group will be asked to receive an exergames intervention for 40 min a day, 3 to 5 days a week, at least 120 min per week, for 4 weeks. The target HRmax should be between 64 and 76% for moderate exercise intensity. The exergames intervention will be facilitated by cardio-respiratory exercise games (Ring Fit Adventure, Mario Tennis Aces, Sports Party, Fit’s Island Cycling, and Aerobic Step) from Nintendo. Patients can choose exergames to achieve a target heart rate according to their preferences. Participants must wear a heart rate monitor during exercise to achieve the target heart rate. In addition to applying heart rate as an objective indicator of exercise intensity, the Borg Rating of Perceived Exertion (RPE) will be applied to measure the individual’s perception of exercise intensity. Participants will be asked to achieve moderate-intensity exercise on a Borg scale of 10 to 13.

##### Intervention group (exergames + ice therapy)

Participants in the intervention group will be asked to receive an exergames intervention for 40 min a day, 3 to 5 days a week, at least 120 min per week, for 4 weeks. The target HRmax should be between 64 and 76% for moderate exercise intensity. Exergames intervention will be facilitated by cardio-respiratory exercise games (Ring Fit Adventure, Mario Tennis Aces, Sports Party, Fit’s Island Cycling, and Aerobic Step) from Nintendo. Participants must wear a heart rate monitor during exercise to achieve the target heart rate. In addition to applying heart rate as an objective indicator of exercise intensity, the Borg Rating of Perceived Exertion (RPE) will be applied to measure the individual’s perception of exercise intensity. Participants will be asked to achieve moderate-intensity exercise on a Borg scale of 10 to 13.

Moreover, the patients will also be treated with ice therapy, utilizing circular localized massage with ice bags made of polyethene plastic on key muscles of the upper and lower extremities that are prone to muscle damage and lactic acid accumulation during exercise as well as on critical joints prone to gout attacks. The duration of ice therapy will be 15 to 20 min after exergames intervention.

To rule out the influence of dietary choices, the researchers will ask all participants to maintain a low-purine diet and abstain from alcohol during the exercise intervention. Moreover, participants will be asked to record their daily intake in diaries. The researcher will regularly monitor and check the diaries to ensure adherence to dietary guidelines.

#### Criteria for discontinuing or modifying allocated interventions {11b}

1. Adverse events: If a participant experiences severe adverse events or side effects directly attributed to the allocated intervention, the intervention may be discontinued or modified as necessary for the participant’s safety. The decision will be based on clinical judgment and in consultation with the study’s principal investigator and relevant medical professionals.
2. Participant withdrawal of consent.
3. The participant fails to comply with the study requirements or guidelines related to the allocated intervention.
4. Changes in the participant’s health status or external factors affecting the study may warrant modification or discontinuation of the allocated intervention.

#### Strategies to improve adherence to interventions {11c}

To improve adherence to the intervention, each participant will receive a transportation subsidy and a free medical screen.

#### Relevant concomitant care permitted or prohibited during the trial {11d}

Participants will not be eligible for the study if they are expected to require additional urate-lowering medication during the trial phase.

#### Provisions for post-trial care {30}

This trial does not provide post-trial care.

### Outcomes {12}

The primary and secondary outcomes of the study will be measured at three time points: baseline (0 weeks), post-intervention (4 weeks), and follow-up (12 weeks). All measurements will be taken at the same location and performed by the same therapist. All data will be collected by a designated investigator and recorded in Microsoft Excel.

#### Primary outcomes

1. Anthropometric parameters: body mass index **(**BMI)
2. Biochemical indicators: the concentration of serum uric acid (SUA)

#### Secondary outcomes

1. Anthropometric parameters: waist circumference (WC), hip circumference (HC), waist-to-hip ratio, range of movement in target join
2. Biochemical indicators: fast blood glucose, total cholesterol (TC), triglyceride, high-density lipoprotein (HDL) cholesterol, low-density lipoprotein (LDL) cholesterol, uric acid (UA), and creatinine
3. Physiological indicators: systolic blood pressure (SBP), diastolic blood pressure (DBP)
4. Subjective indicators: pain intensity (24 h after exercise, measurement tool: visual analogue scale (VAS)), gout-specific quality of life (measurement tool: The Gout Impact Scale (GIS)), kinesiophobia (measurement tool: The Tampa Scale of Kinesiophobia (TSK)), positive and negative affect of exercise (measurement tool: Positive and Negative Affect Schedule (PANAS))
5. Adverse effect: rate of gout flare

### Participant timeline {13}

**Fig. 2:**
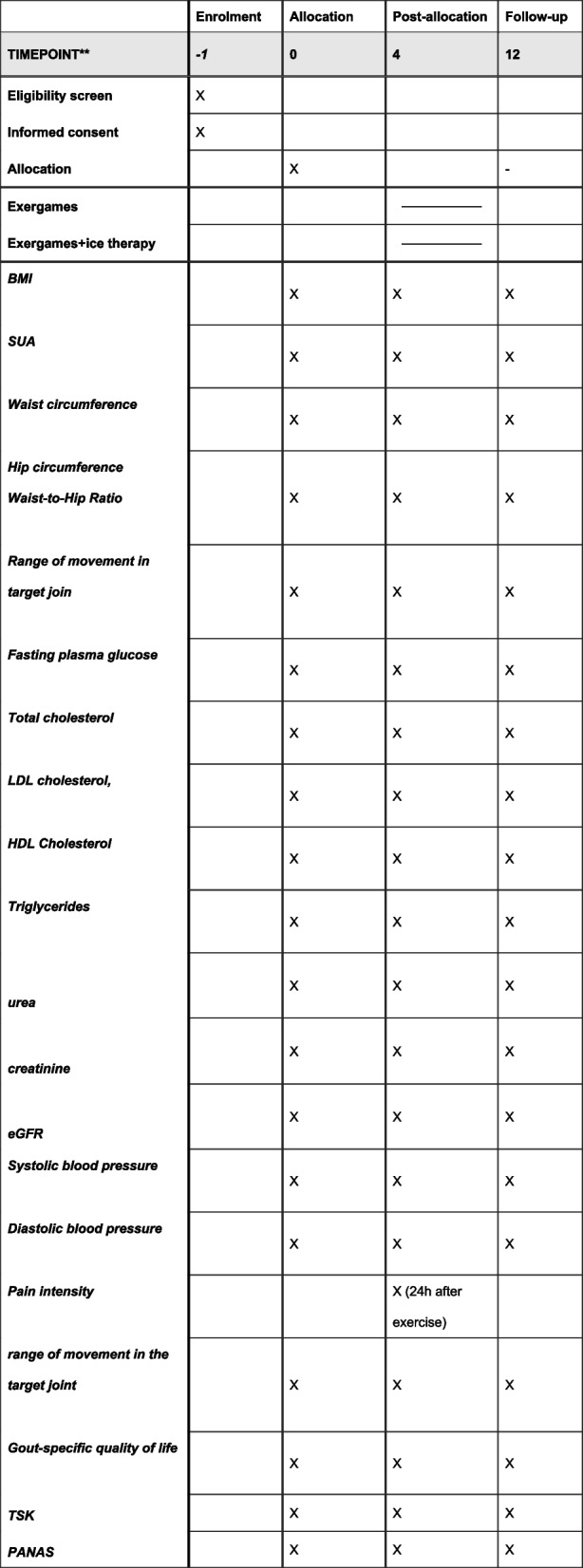
Schedule of enrollment, interventions, and assessments

The schedule of enrollment, interventions, and assessments is shown in Fig. 2.

### Sample size {14}

The sample size was calculated using GPower. This study is a randomized controlled trial with an equal number of subjects in the intervention and control groups. The primary outcome measure was the serum uric acid concentration. According to previous studies [21], the serum uric acid concentration of patients in the control group was 420 ± 100 µmol/L. The effect size was set to 0.25, bilateral *α* = 0.05 was set, and the power was 80%; at least 14 patients will be included in each group, making the sample size *N* = 28. Considering a 10% follow-up dropout rate, at least 30 patients will be included, with a minimum of 15 patients in each group.

### Recruitment {15}

Participants will be recruited at ADMI between June 2023 and September 2024. Researchers will access the medical records of gout patients within the ADMI system. Once eligibility is confirmed, potential participants will be contacted by phone to inquire about their willingness to participate in the study. Additionally, clinic physicians will distribute study advertisements to eligible gout patients.

## Assignment of interventions: allocation

### Sequence generation {16a}

Participants will be randomly assigned to either the control group and intervention group in a 1:1 ratio by a researcher using computer-generated random numbers table. Each participant will receive a unique number, which will then be matched with a corresponding group assignment (either control or intervention) based on the random number table. The researcher will prepare 30 sealed envelopes and place randomly generated serial numbers inside them in order.

### Concealment mechanism {16b}

After confirming that the participants are qualified, the envelopes are opened sequentially, and the participants are assigned to their respective groups. Those responsible for generating the random assignments will not be participating in the trial.

### Implementation {16c}

The statistician independent of the trial will generate the allocation sequence, physicians will enroll participants, and the nurse will assign participants to interventions.

## Assignment of interventions: blinding

### Who will be blinded {17a}

This is a single-blind randomized controlled trial in which the researchers responsible for patient assessment and data collection will be unaware of the intervention allocations.

### Procedure for unblinding if needed {17b}

This study will not involve unblinding procedures.

## Data collection and management

### Plans for assessment and collection of outcomes {18a}

An outcomes assessor will measure the results before and after the intervention, followed by a 12-week follow-up. The study intervention will be conducted at AMDI, USM, and patients will be registered at the exercise clinic at AMDI, USM.

### Plans to promote participant retention and complete follow-up {18b}

Provide participants with a comprehensive overview of the study timeline and emphasize the importance of their continued participation. Moreover, regular reminders will be sent to participants via email, text messages, and phone calls to facilitate timely scheduling and encourage their attendance at upcoming follow-up assessments.

### Data management {19}

All data forms and original laboratory records collected during the study will be stored in lockable filing cabinets within the office in AMDI. Additionally, digital data will be stored on the researcher’s official work desktop computer, accessible only through a password-protected login. All data will be maintained for 7 years.

### Confidentiality {27}

All study data will be securely stored in password-protected electronic databases, accessible only to authorized personnel involved in the research. Personal identifiers will be replaced with unique study identification codes to anonymize participant information. Physical documents containing participant data will be stored in locked cabinets in a restricted-access area.

### Plans for collection, laboratory evaluation, and storage of biological specimens for genetic or molecular analysis in this trial/future use {33}

The excess/leftover blood specimens will be stored for 2 years and discarded after this period. The blood specimen will not be used for other research during the storage period.

## Statistical methods

### Statistical methods for primary and secondary outcomes {20a}

All data will be analyzed using SPSS version 29. One-way ANOVA will be used to evaluate whether there were significant differences in age, gender, and symptom severity of gout between the intervention and control groups. Two-way repeated measures ANOVA will be used to analyze differences in outcomes before and after treatment. The significance level will be set at *p* < 0.05. If some patients are unable to complete the experiment due to uncontrollable factors or if they violate the inclusion criteria during the study, these patients will be excluded from the analysis.

### Interim analyses {21b}

The data and safety monitoring committee will be responsible for monitoring the safety and efficacy of this study and is authorized to make decisions regarding the termination of the trial.

### Methods for additional analyses (e.g., subgroup analyses) {20b}

This study will pre-specify subgroups for the comparison of treatment effects. These subgroups are based on known factors that may influence treatment outcomes in gout patients, including patient age, prior treatments, and the severity of gout.

### Methods in analysis to handle protocol non-adherence and any statistical methods to handle missing data {20c}

In this study, intention-to-treat (ITT) analysis will be employed to assess the trial policy regarding the treatment received by allocated participants, while per-protocol (PP) analysis will be utilized to exclude or scrutinize participants deviating from the treatment protocol. This study anticipates minimal or no occurrence of missing outcome data, as the trial will not be terminated before all data is collected.

### Plans to give access to the full protocol, participant-level data, and statistical code {31c}

All data relevant to the trial will be made publicly accessible following reasonable requests upon the completion of the trial.

## Oversight and monitoring

### Composition of the coordinating center and trial steering committee {5d}

The trial steering committee will consist of an experienced clinical expert in gout and an exercise rehabilitation expert. They will serve as an independent team to oversee the conduct of the trial.

### Composition of the data monitoring committee, its role, and reporting structure {21a}

The data monitoring committee is composed of an exercise rehabilitation expert and a statistician who are independent and free from any conflicts of interest. They are responsible for scrutinizing the integrity and safety of the data.

### Adverse event reporting and harms {22}

Due to the intervention being safe and non-invasive, there is a lower risk of developing adverse events in this study. Patients will be withdrawn from the trial if they experience any adverse event related to the intervention, such as exercise injuries, falls, chest pain, dyspnea, or hypoglycemia. Furthermore, all adverse events will be recorded and reported in the study results.

### Frequency and plans for auditing trial conduct {23}

An independent data monitoring committee, separate from the investigators and sponsors, will conduct audits of the trial at the 25%, 50%, and 75% stages of trial progression.

### Plans for communicating important protocol amendments to relevant parties (e.g., trial participants, ethical committees) {25}

All revisions to the protocol require approval from the Human Research Ethics Committee at USM. Once the revised protocol is approved, we will inform the participants and the involved researchers. We will also revise and update the protocol in the clinical trial registry. Any deviations from the protocol will be fully documented using a breach report form and submitted to Human Research Ethics Committee at USM.

### Dissemination plans {31a}

The results of this study will be published in an international, peer-reviewed journal, and the raw data will also be made publicly available through publication on an open data repository. Moreover, the results of the study will be reported in the Chinese Clinical Trial Registry and disseminated through patient and stakeholder workshops as well as relevant international conferences.

## Discussion

According to Jablonski et al. [9], low- to moderate-intensity exercise prevents inflammatory responses and decreased tissue swelling in a mouse model of acute gout. A cohort study conducted by McCormick et al. [22] indicates that addressing obesity is key for men with gout to benefit from other gout management approaches; obesity is also an internationally recognized modifiable risk factor for recurrent gout attacks [7]. Therefore, maintaining a non-obese weight and losing weight is a key factor in gout treatment [23].

It is generally acknowledged that neglecting exercise is one of the most prominent risk factors for increased BMI [24]. Personalized exercise rehabilitation interventions are effective for weight loss [8]. According to Nishida et al. [25], moderate-intensity exercise exhibited a negative correlation with serum uric acid levels in obese Japanese men. A large cross-sectional study based on the National Health and Nutrition Examination Survey (NHANES) in the United States demonstrated a non-linear relationship between exercise and SUA levels [26].

The British Society for Rheumatology Guideline for the Management of Gout suggests that ice could be an adjunct to acute gout treatment as it could effectively reduce pain in gout patients [1]. Schlesinger et al. [27] conducted a study to evaluate the effect of topical ice therapy on patients suffering from acute gout. The research result indicates that ice therapy significantly reduces pain. The application of post-exercise ice therapy can reduce tissue temperature and blood flow, thereby alleviating cardiovascular stress, eliminating muscle metabolic by-products, reducing exercise-induced symptoms, and accelerating post-exercise recovery [28]. Numerous studies have shown that ice therapy can effectively relieve DOMS at 24 h after exercise [29–33].

Exergames technology has been widely recognized in rehabilitation and has achieved positive results [34]. Fitness games used for therapeutic purposes, also known as serious games, can provide patients with an immersive experience that allows them to focus on training and achieve more optimal results in challenging and competitive programs [35]. Exergames typically provide light to moderate exercise training to help users burn calories. Studies have demonstrated the beneficial effects of exergames as an aid in weight loss [36]. Moreover, few studies report adverse events in exergames, and the safety of exergames is widely recognized [37]. However, the long-term effects of exergames need to be supplemented and verified by future research [36].

To our knowledge, no study has investigated the effect of exergames and ice therapy among gout patients. However, research evidence supports that exergames can improve persistence and significantly reduce BMI. Ice therapy can increase the patient’s adherence to exercise by reducing symptoms and joint responses after exercise, thereby assisting in better reducing BMI. Therefore, this study aims to examine the combined effect of exergames and ice therapy in patients with gout in Pulau Pinang. This study is expected to demonstrate that exergames combined with ice therapy are more effective in gout management compared to exergames alone. The outcome of this study could be used as guidelines for choosing the right exercise program to improve the health status of the gout population. This study would also benefit patients with gout by highlighting any gaps between their awareness and the correct concepts of gout management, encouraging them to prioritize exercise. The findings of this study may help healthcare practitioners re-evaluate gout treatments to identify and address deficiencies in current approaches to gout management, and establish a new program and perspective for gout management.

However, this study has some major scopes. Since no studies have reported the optimal exercise intensity and frequency for patients with gout, this study does not account for the potential effects of different intensities and frequencies of exercise. Moreover, as this study only included patients from Pulau Pinang, the findings may lack universality.

## Trial status

The protocol version number is 1.0 and the date is 17 March 2023. This study commenced participant recruitment at ADMI in June 2023, with an anticipated completion of data collection by September 2024.

## Data Availability

All data generated or analyzed during this study will be included in the future published article and its supplementary information files. All data required by this protocol will be made available upon request.

## Abbreviations

MSU: Monosodium urate crystals (MSU)
ADMI: The Advanced Medical and Dental Institute
USM: Universiti Sains Malaysia
ACR: The American College of Rheumatology
EULAR: The European League of Associations for Rheumatology
ULT: Urate-lowering therapy
HDL: High-density lipoprotein
LDL: Low-density lipoprotein
eGFR: Estimated glomerular filtration rate
RPE: Borg Rating of Perceived Exertion
PANAS-SF: Positive and Negative Affect Schedule
BMI: Body mass index
SUA: Serum uric acid
VAS: Visual analogue scale
GIS: Gout Impact Scale
TSK: Tampa Scale of Kinesiophobia
PANAS: Positive and Negative Affect Schedule
ITT: Intention-to-treat
PP: Per-protocol
